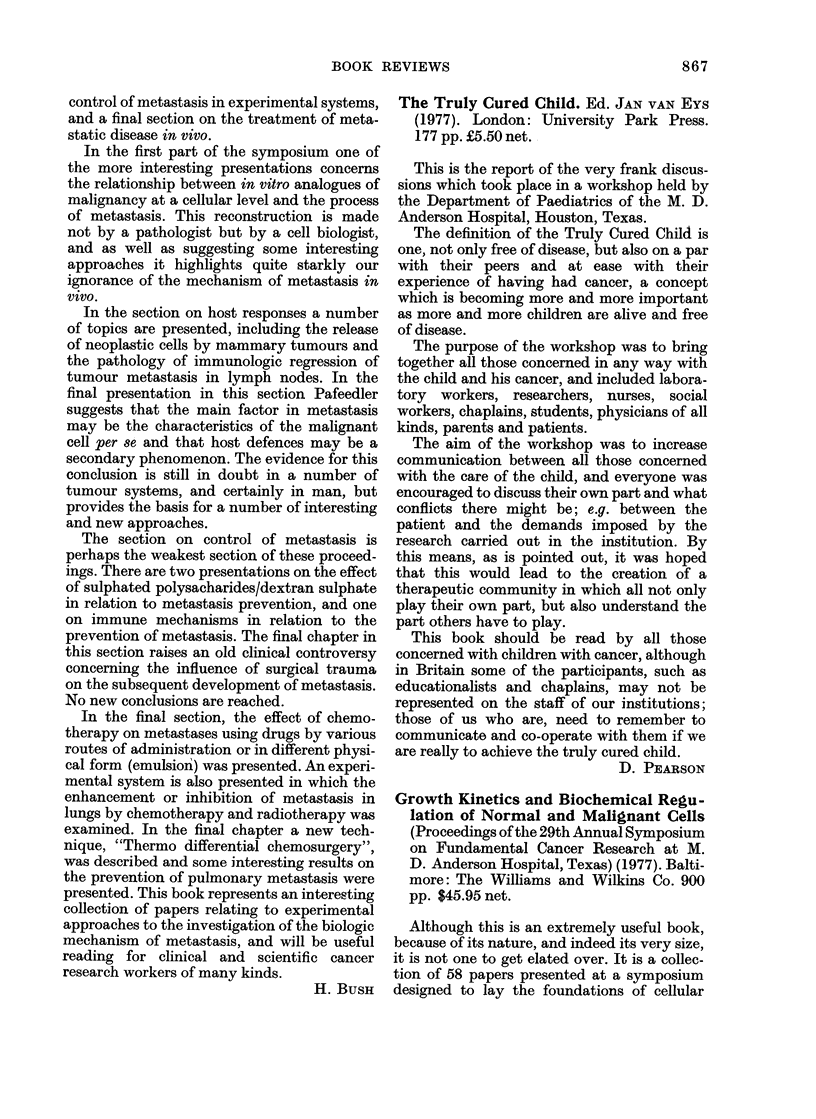# The Truly Cured Child

**Published:** 1978-05

**Authors:** D. Pearson


**The Truly Cured Child.** Ed. Jan van Eys (1977). London: University Park Press. 177 pp. £5.50 net.

This is the report of the very frank discussions which took place in a workshop held by the Department of Paediatrics of the M. D. Anderson Hospital, Houston, Texas.

The definition of the Truly Cured Child is one, not only free of disease, but also on a par with their peers and at ease with their experience of having had cancer, a concept which is becoming more and more important as more and more children are alive and free of disease.

The purpose of the workshop was to bring together all those concerned in any way with the child and his cancer, and included laboratory workers, researchers, nurses, social workers, chaplains, students, physicians of all kinds, parents and patients.

The aim of the workshop was to increase communication between all those concerned with the care of the child, and everyone was encouraged to discuss their own part and what conflicts there might be; *e.g.* between the patient and the demands imposed by the research carried out in the institution. By this means, as is pointed out, it was hoped that this would lead to the creation of a therapeutic community in which all not only play their own part, but also understand the part others have to play.

This book should be read by all those concerned with children with cancer, although in Britain some of the participants, such as educationalists and chaplains, may not be represented on the staff of our institutions; those of us who are, need to remember to communicate and co-operate with them if we are really to achieve the truly cured child.

D. Pearson